# Supplementary material for: A Critical View of the Application of the APEX Software (Aqueous Photochemistry of Environmentally-Occurring Xenobiotics) to Predict Photoreaction Kinetics in Surface Freshwaters
Source: Molecules. 2019 Dec 18;25(1):9. doi: 10.3390/molecules25010009 (PMC7017383; doi:10.3390/molecules25010009)
Supplement: Supplementary file 1 [file molecules-25-00009-s001.zip › 3.2.4_gcc-4.4.0.zip!/3.2.4_gcc-4.4.0/3.2.4_gcc-4.4.0/doc/octave/html/liboctave/Linear-Constraints.html]

Linear Constraints - Octave C++ Classes


Next: Nonlinear Constraints,
Previous: Bounds,
Up: Optimization


---

### 9.3 Linear Constraints

— : **LinConst** (void)  
— : **LinConst** (int nclin, int nx)  
— : **LinConst** (int nclin\_eq, int nclin\_ineq, int nx)  
— : **LinConst** (const ColumnVector &lb, const Matrix &A, const ColumnVector &ub)  
— : **LinConst** (const Matrix &A\_eq, const ColumnVector &b\_eq, const Matrix &A\_ineq, const ColumnVector &b\_ineq)  
— : **LinConst** (const LinConst &a)

— : LinConst& **operator =** (const LinConst &a)

— : LinConst& **resize** (int nclin, int n)

— : Matrix **constraint\_matrix** (void) const;

— : LinConst& **set\_constraint\_matrix** (const Matrix &A)

— : Matrix **eq\_constraint\_matrix** (void) const;  
— : Matrix **ineq\_constraint\_matrix** (void) const;

— : ColumnVector **eq\_constraint\_vector** (void) const;  
— : ColumnVector **ineq\_constraint\_vector** (void) const;

— : ostream& **operator <<** (ostream &os, const LinConst &b)
